# Concentration of Trace Elements in Patients with Aortic Stenosis and Coexisting Coronary Artery Disease: A Pilot Study

**DOI:** 10.3390/jcm15010008

**Published:** 2025-12-19

**Authors:** Anna Olasińska-Wiśniewska, Tomasz Urbanowicz, Marcin Misterski, Marek Grygier, Antoni F. Araszkiewicz, Filip Wojewódzki, Sebastian Stefaniak, Paweł Marcinkowski, Ilona Kauf, Marek Jemielity, Anetta Hanć

**Affiliations:** 1 Department of Cardiac Surgery and Transplantology, Poznan University of Medical Sciences, 61-848 Poznan, Poland; turbanowicz@ump.edu.pl (T.U.); mister@poczta.onet.pl (M.M.); sebastian.stefaniak@usk.poznan.pl (S.S.); pawel.marcinkowski@usk.poznan.pl (P.M.); ilona.kauf@usk.poznan.pl (I.K.); mjemielity@poczta.onet.pl (M.J.); 2 First Department of Cardiology, Poznan University of Medical Sciences, 61-848 Poznan, Poland; marek.grygier@usk.poznan.pl; 3 Student Scientific Society, Poznan University of Medical Sciences, 61-848 Poznan, Poland; antek.araszkiewicz@gmail.com; 4 Biogeochemistry Research Unit, Faculty of Geographical and Geological Sciences, Adam Mickiewicz University, 61-680 Poznan, Poland; filip.wojewodzki@amu.edu.pl; 5 Department of Trace Analysis, Faculty of Chemistry, Adam Mickiewicz University, 61-680 Poznan, Poland; anetta.hanc@amu.edu.pl

**Keywords:** trace elements, aortic stenosis, coronary artery disease

## Abstract

**Background/Objectives:** Coronary artery disease (CAD) and aortic stenosis (AS) frequently coexist and share similar pathophysiological pathways, including inflammation, lipid deposition, and extracellular matrix remodeling. Trace elements are involved in cellular and physiological processes, playing regulatory and signaling roles. Their concentrations may be altered in various pathological conditions. The aim of our study was to compare trace metal concentrations in patients with severe aortic stenosis with and without coexisting coronary artery disease. **Methods:** In 53 patients (25 male, 47.2%, median age of 78 (75–81) years) with severe aortic stenosis, CAD coexistence and progression were analyzed based on the most recent coronary angiography report and history of revascularization. Blood samples for trace element analysis were collected prior to the implantation of the prosthesis, from the peripheral artery and by the pigtail catheter at the aortic root. **Results:** Twenty-six patients presented any degree of CAD, and were further differentiated into more advanced disease stages. The analysis found that patients with CAD had lower median concentrations of aluminum and calcium in the peripheral blood, and manganese and selenium in the aorta. Furthermore, in most advanced CAD patients, the concentration of magnesium, calcium, nickel, and copper in peripheral blood, along with chromium and selenium in aortic blood, was found to be lower compared to non-CAD patients. Lower selenium in aortic blood samples was predictive of an advanced stage of CAD. **Conclusions:** Patients with severe aortic stenosis and coexisting CAD present significantly lower blood concentrations of trace elements compared to those with the isolated disease.

## 1. Introduction

Aortic stenosis (AS) is the most common heart valve disease, and coronary artery disease (CAD) is the leading cause of cardiovascular mortality in developed countries. Both diseases frequently coexist and share similar pathophysiological pathways and risk factors, which increases the risk profile and complicates management strategies. Common risk factors include male sex, diabetes mellitus (DM), arterial hypertension (HA), hyperlipidemia, obesity, chronic kidney disease, sedentary lifestyle, and smoking. However, there are also several differences between the two diseases. While AS has a progressive character, which gradually impairs leaflet mobility, with calcification, left ventricular hypertrophy, and failure, CAD follows a nonlinear step-line pattern with atherosclerotic plaque ruptures [1]. In AS, the initial endothelial injury is followed by immune cell infiltration, lipid deposition, fibroblastic and osteoblastic differentiation of valvular interstitial cells, and calcification [2]. The hallmark of CAD is atherosclerotic plaque formation, initiated by subendothelial deposition of lipid-loaded macrophages with oxidized low-density lipoproteins (LDL) called foam cells. The inclusion of immune cells, such as T cells and cytokines, further aggravates the inflammatory milieu. Vascular smooth muscle cells proliferate and form a fibrous cap. The release of calcifying extracellular vesicles and apoptosis leads to the formation of microcalcifications. Plaque stability is related to the type of calcification [3]. Stable plaques are characterized by macrocalcifications and a thin collagen-rich extracellular matrix, while unstable plaques tend to include microcalcifications and a thick fibrous cap [4].

In the coexistence of diseases, severe CAD is associated with worse outcomes in AS patients [5]. The coexistence of AS and CAD may require adjustments in management, particularly in unexpected cases. The routine use of biomarkers for disease detection would be important in diagnosis and therapy planning [6]. The timing of subsequent therapeutic stages is particularly crucial in patients qualified for transcatheter procedures, due to technical challenges and the significance of AS for heart muscle hypertrophy and remodeling.

Essential trace metals, such as iron, zinc, or copper, are involved in vital cellular and physiological processes, performing catalytic, regulatory, and signaling roles [7,8]. However, their alterations are related to inflammation [9,10]. The association of metal and trace elements exposure with cardiovascular morbidity has been postulated [11,12,13]. The disparities in concentrations between calcified and healthy aortic valves were shown in a study by Tomasek et al. [14]. While atherosclerosis and aortic stenosis used to be analyzed as discrete disorders, in daily practice, they often coexist. The aim of our study was to compare trace metal concentrations in patients with severe aortic stenosis with and without accompanying CAD.

## 2. Materials and Methods

### 2.1. Study Patients

This prospective observational study enrolled 53 consecutive patients with symptomatic (class II—IV of the New York Heart Association classification (NYHA)) severe aortic stenosis who underwent TAVI at the heart team’s discretion. Study exclusion criteria included active neoplastic diseases and the use of general anesthesia. No patients used a restrictive diet.

### 2.2. Methods

Demographic and clinical data were collected during the qualification process and at admission for the procedure.

Arterial hypertension (HA) was recognized in patients with a systolic pressure of 140 mm Hg or a diastolic pressure of 90 mm Hg or a history of use of antihypertensive drugs. The diagnosis of diabetes mellitus (DM) or impaired glucose tolerance (IGT) was documented based on the history and the use of medication. Chronic obstructive pulmonary disease (COPD) was recorded based on the history and the use of inhaled treatment. Smoking history was collected.

Blood samples for standard biochemical and hematological analyses were collected at admission. Blood samples for trace metal analysis were collected during the procedure before prosthesis implantation. Among the metal elements lithium (Li), magnesium (Mg), aluminum (Al), titanium (Ti), vanadium (V), chromium (Cr), manganese (Mn), iron (Fe), cobalt (Co), nickel (Ni), copper (Cu), zinc (Zn), arsenic (As), selenium (Se), rubidium (Rb), strontium (Sr), molybdenum (Mo), cadmium (Cd), cesium (Cs), barium (Ba), lead (Pb), as well as other elements such as calcium (Ca), were analyzed.

Pre-procedural echocardiography was performed by the same group of experienced echocardiographers using a standardized protocol based on current recommendations for assessing aortic stenosis. They assessed aortic stenosis severity with peak and mean transvalvular gradients and aortic valve area, aortic insufficiency, left ventricular contractility with left ventricular ejection fraction, and other valvular dysfunctions.

TAVI was performed in the hybrid room by the same team of experienced operators under fluoroscopic and echocardiographic guidance, using transfemoral access. Local anesthesia was used in all patients. Self-expandable prostheses were implanted, and the selection of procedural features (device, implantation technique, etc.) was at the operators’ discretion. The blood samples for trace elements analysis were collected before the prosthesis implantation. “Peripheral” arterial blood samples were collected before the procedure by the anesthesiologist team, and the “aortic” blood samples were obtained from the pigtail catheter at the aortic root.

Coronary angiography was performed in all patients to reveal the coronary atherosclerosis status. In case of significant coronary artery stenosis in the proximal segment, percutaneous coronary intervention (PCI) was performed. Moreover, the history of any percutaneous coronary angioplasty (PCI) or coronary artery bypass grafting (CABG), and history of any coronary intervention within the preceding year, were collected.

Based on the coronary angiogram, patients were divided into a subgroup without any coronary changes (isolated AS—non-CAD patients) and patients with CAD (CAD-patients). However, our CAD subgroup included patients at different degrees of CAD, both currently and in the past, which could have potentially influenced heart muscle function and trace element concentration. Therefore, in the next step, we excluded patients without significant CAD and differentiated a subgroup of patients with >50% for the left main (LM) and >70% for other coronary branches or a history of revascularization (any revascularization or LM > 50% or >other coronary artery stenosis > 70%). Moreover, we also differentiated patients with any history of revascularization (any revascularization). We then compared trace element concentration in the non-CAD group with concentrations in the relevant subgroups.

### 2.3. Elemental Analysis

Blood samples were carefully prepared for elemental analysis using inductively coupled plasma mass spectrometry (ICP-MS). Approximately 1 mL of blood was mixed with 3 mL of 65% nitric acid and 0.5 mL of 30% hydrogen peroxide for digestion. The digestion process included two phases: a 3 h pre-mineralization followed by mineralization in a closed vessel system. Samples were mineralized using a microwave digestion system (EthoS One, Milestone, Sorisole, Italy). The heating program consisted of a 20 min temperature ramp to 220 °C, maintained at 220 °C for 30 min, followed by 20 min of controlled cooling. The system operated at a power of 1500 W. After digestion, the samples were diluted to a final volume of 50 mL using distilled water (Direct-Q-3 UV, Merck, Darmstadt, Germany). Certified reference materials, Seronorm Trace Elements in whole blood, level 1 and level 2, were processed identically to evaluate measurement accuracy.

Elemental analysis was performed using an inductively coupled plasma mass spectrometer (ICP-MS 7100x, Agilent, Santa Clara, CA, USA). Instrument settings were optimized automatically with Tuning Solution (Agilent, Santa Clara, CA, USA). To minimize spectral interferences, the helium collision mode was used alongside an internal standard solution containing 10 µg/L rhodium and terbium. Calibration standards were prepared by diluting a 10 mg/L multi-element standard solution (ICP Standard, Perkin Elmer, Darmstadt, Germany) across a range of 0.01–100 µg/L. High-purity argon (99.999%) was employed as the nebulizer, auxiliary, and plasma gas (Messer, Chorzów, Poland).

Method validation was performed using the Seronorm Trace elements in whole blood, level 1 and level 2 certified reference materials, with performance assessed by parameters including linearity, precision, limit of detection (LOD), and accuracy. Calibration curves showed excellent linearity with correlation coefficients (R) above 0.9996 for all analytes. LODs, calculated as 3.3 times the standard deviation of blank samples divided by the calibration curve slope, ranged from 0.002 µg/g for Cd to 0.214 µg/g for Fe. Precision, expressed as coefficient of variation (CV), ranged between 1.2% and 3.4%. Accuracy, evaluated by recovery of reference materials, ranged from 96% to 102%.

### 2.4. Ethics Approval

The study was approved by the institutional Ethics Committee (No 272/2021 dated 8 April 2021); written informed consent was obtained from each participant, and the study protocol conformed to the ethical guidelines of the 1975 Declaration of Helsinki.

### 2.5. Statistical Analysis

Data normality was assessed using the Shapiro–Wilk test. Continuous variables were reported as mean ± standard deviation (SD) or median (interquartile range (IQR)) and compared using t-tests or Mann–Whitney U tests, as appropriate. Categorical variables were compared using Fisher’s exact tests. Trace elements were included in the multivariable analysis to reveal their potential for CAD prediction. Statistical significance was set at *p* < 0.05. Analyses were performed using JASP software (JASP Team, 2020, Version 0.13.1).

## 3. Results

### 3.1. Study Group

The study group included 53 patients (25 male (47.2%), median age of 78 (75–81) years). They were burdened with co-morbidity, including 25 (47.2%) with DM or impaired glucose tolerance (IGT), 47 (88.7%) with HA, 5 (9.4%) with chronic obstructive pulmonary disease (COPD), and 13 (24.5%) with atrial fibrillation. Patients’ demographic and clinical data are presented in Table 1.

Twenty-six patients presented with CAD. Fifteen of them (57.7%) had a history of PCI (*n* = 14) or CABG (*n* = 1), and ten (38.5%) underwent PCI within the year preceding TAVI. Six patients (23.1%) had an acute myocardial infarction.

During the last coronary angiography before TAVI, four patients presented with at least 50% left main stenosis, seven patients presented with at least 70% left anterior descending artery stenosis, six patients presented with over 70% circumflex artery stenosis, and six patients had over 70% stenosis in the right coronary artery. Of these, ten patients underwent simultaneous PCI.

### 3.2. Trace Elements Concentration Analysis

The trace metal concentrations were analyzed in the blood samples collected from the peripheral artery (P) and in proximity to the aortic valve (A) (Table 2). The concentrations were compared between CAD and non-CAD patients, revealing lower median peripheral blood Al and Ca, and aortic Mn and Se levels in CAD patients. Following this, we differentiated patients with more advanced CAD. Thus, we compared patients without CAD to patients who underwent any revascularization in their history, and showed lower concentrations of peripheral blood Ca, Ni, Cu, and aortic blood Ca, Cr, Cu, Se, and Mo in the latter group. In the group comprising patients with coronary artery stenosis over 70% or LM > 50% or previous revascularization, peripheral blood Mg, Al, Ca, Ni, Cu, Se, and aortic blood Cr, Cu, Se, and Mo had lower concentrations than non-CAD patients.

Furthermore, the value of trace elements in predicting CAD in patients with severe AS was evaluated in the multivariable analysis, separately for aortic and peripheral samples. Only aortic Ca (*p* = 0.029) and Se (*p* = 0.027) concentrations were predictive (AUC 0.867, with sensitivity of 33.3% and specificity of 88%) for patients with advanced CAD who ever required coronary revascularization, and Se concentrations (*p* = 0.018, AUC 0.767, sensitivity of 45.5%, specificity of 92%) for patients with advanced CAD who ever required coronary revascularization and/or presented with at least 60% stenosis at the qualification timepoint.

## 4. Discussion

Our study revealed that patients with isolated aortic valve disease and patients with concomitant CAD share similarities in blood concentrations of the majority of trace elements; however, there are significant differences in blood concentrations of Al, Ca, Cr, Mn, Ni, Cu, Se, and Mo. Particularly, serum Se concentration differentiated patients with advanced CAD.

Recent studies emphasize distinct cellular responses in the background of both diseases. AS prominently features extracellular matrix calcification driven by osteogenic differentiation of valvular interstitial cells, while CAD is marked by smooth muscle cell proliferation and inflammatory plaque instability [15]. Mechanical stress due to altered hemodynamics contributes to endothelial dysfunction in both diseases, but manifests differently due to anatomical and functional differences [2,4]. Both diseases often occur together and share common underlying mechanisms, such as chronic inflammation and mineral imbalance. Recent epidemiological and imaging research has found that many patients with severe AS also have CAD, with similar risk factors and disease progression patterns [16]. Our results, which reveal deficiencies in trace elements, support the observations that systemic and local metabolic disturbances collectively contribute to the development of both conditions, aligning with observations from large population studies and tissue analyses.

Our analysis comprises patients with the advanced stage of AS and different scenarios of coronary artery involvement. Notably, all patients present cardiovascular disease, with severe aortic stenosis as the dominant one. However, we distinguished patients at different stages of CAD. First, we selected all patients who presented any degree of coronary atherosclerosis, with coronary stenosis of at least 30% and/or any coronary intervention in history. In the next steps, we analyzed patients who ever presented the need for revascularization and those with previous revascularization and coronary stenosis of over 60%. Using these selections, we aimed to assess trace element concentrations in patients with less severe CAD compared to those with severely advanced disease.

Moreover, we collected blood samples from the peripheral artery and from the proximity to the aortic valve. This maneuver was implemented to determine possible differences in trace element concentration at the nearest point to the pathologically changed tissue. Since we lacked spatial trace element analysis in tissues, we tried to compare potential differences in different (peripheral vs. valvular) regions. The most interesting finding was the abnormality in selenium levels in the aortic samples.

Our analysis, compared to large health surveys [17,18], showed that concentrations of zinc, copper, selenium, chrome, and molybdenum in the whole group and particularly in the CAD subgroup, were lower or at low levels. Aortic stenosis combined with CAD was associated with a more significant trace element deficiency.

Trace metals are essential elements of vital patterns. They are involved in a variety of functions, including catalytic, structural, and regulatory roles, interacting with enzymes, pro-hormones, pre-secretory granules, and biological membranes [19]. Their excess or deficiency may result in severe bodily malfunction or be an indicator of pathological disfunctions and imbalanced shifts between tissues. The significance of trace elements imbalance has been underlined in several disorders [20], including diabetes [21], celiac disease [22], and cardiovascular pathologies [23]. The trace elements status may influence the atherosclerosis development and burden in unstable mode. Notably, certain elements, such as magnesium, zinc, and selenium, play a protective role, while others, including iron and copper, exhibit a multifaceted complex function in atherosclerotic pathogenesis [24].

Out of dozens of essential and toxic elements available for analysis, some have a particular impact on cardiovascular health; therefore, we focused on selected ones in our study.

Aortic stenosis is considered a complex fibrocalcific valvulopathy that results from the interplay between chronic inflammatory system activation, oxidative stressors activation, combined with extracellular matrix (ECM) remodeling, and finally osteogenic reprogramming in the valvular tissue [25]. Trace element-dependent metalloenzymes are considered cofactors of these processes [14]. Dysregulated homeostasis of zinc, copper, chromium, and selenium may be involved in pathogenic cascades that lead to the progression of aortic stenosis [23].

Zinc is an essential trace element that plays a crucial role in maintaining cellular integrity, facilitating protein synthesis, and regulating nucleic acid metabolism. This trace element is considered an obligate cofactor for matrix metalloproteinases (MMPs), whose role in ECM architecture and collagen crosslinking is postulated [26]. Zinc dysregulation of hemostasis may occur bidirectionally, as zinc deficiency promotes pathologic collagen turnover and fibrotic stiffening, but aberrant ECM accumulation is linked with zinc accumulation [27]. Both pathological states accelerate aortic disease progression [28]. It regulates the expression and activation of transcription factors, enzymes, channels, and growth factors. It is implemented in immunity and protection against free radicals, presenting antioxidant and anti-inflammatory effects [22,29], improving the antioxidant capacity of cells. Low levels were related to atherosclerosis progression [30,31]. Zinc deficiency increases expression of bone morphogenetic proteins (BMP2, BMP4) [32]. Its deficiency creates a pro-calcific milieu.

Copper has an important role in energy metabolism, mitochondrial respiration, and antioxidant activity, serving as an enzyme cofactor [22,33]. Its imbalance can trigger cellular toxicity, impair lipid metabolism, resulting in oxidative stress, mitochondrial, and endothelial cell damage [14]. The activity of superoxide dismutase 1 can be reduced, accompanied by an increase in oxidized LDL formation, due to low copper levels [34,35]. Oxidative stress plays a significant role in cardiovascular pathology. High copper levels were associated with the risk of coronary artery disease [36,37] and aortic stenosis [12], cardiovascular morbidity, including stroke, myocardial infarction, and coronary artery disease mortality [38,39]. Our previous analysis proved an association between copper levels and inflammatory induction in CAD [9]. However, some analyses did not confirm the relationship between copper content in body samples and CAD [40], suggesting ethnic differences in copper concentration [41].

Chromium, a heavy metal, serves as a component and activator of enzymes in redox reactions and lipid metabolism [42]. A low toenail chrome concentration was associated with increased risk of myocardial infarction [43]. Chromium’s role is more indirect than that of other elements, but it is still mechanistically relevant, as Cr potentiates insulin receptor sensitivity and its disturbances in glucose metabolism accelerate valvular calcification. Chromium dysregulation affects endothelial cell function by promoting lipid infiltration in the valve and increasing glycation end-product (AGE) formation [44,45]. The association between chromium and lower nitric oxide (NO) production has been noticed [46]. Moreover, the Cr insufficiency increases leukocyte adhesion and induces enhanced oxidative stress in the valvular microenvironment [47].

Selenium is an essential trace element; however, its role is less commonly discussed in the literature. Its role in two central systems protecting valvular tissue against hydrogen peroxide (H_2_O_2_) and lipid hydroperoxides via glutathione peroxidases (GPx1, GPx3) and thioredoxin reductases is postulated [48,49]. Optimal selenium status suppresses redox-sensitive signaling pathways, including p38 MAPK, ERK1/2, and JNK [50,51]. The role of Se in immune system modulation is based on its involvement in macrophage-driven inflammatory signaling, NF-κB activation, and release of pro-calcific cytokines such as IL-6, TNF-α, and IL-1β [52,53,54]. Enzymes containing Se regulate antioxidant status and immune response. Reduced Se-related glutathione peroxidase activity is implicated in the generation of toxic lipid peroxides, endothelial dysfunction, and arterial stiffness [55]. Total Se in selenoproteins was positively correlated with increased 10-year relative risk of cardiovascular disease [56].

According to literature reports, concentrations of elements such as Fe, As, Ca, Co, Cr, Mg, P, Pb, Se, Sn, Sr, and Zn are higher in stenotic aortic valve tissue compared to controls [10,57]. These observations, together with our results presenting low serum concentrations, may result from the higher infiltration in the diseased tissues and may also reflect the advancement of the disease. Lis et al. [58] found that accumulation of calcium and phosphorus in calcified areas of valves was accompanied by enhanced concentrations of strontium and zinc. Al-Taesh et al. [59] investigated the relationship between AS and serum levels of Fe, Zn, Se, and Cu. They reported higher serum Cu and lower serum Se, Zn, and Fe concentrations in the diseased groups compared to healthy patients.

In our analysis, the zinc concentration in both peripheral and aortic samples did not differ between subgroups. Its deficiency was observed in all subgroups, indicating its strong association with both coronary atherosclerosis and valve calcification processes. It may express the relationship between deficiency and disease progression, or exhibit as zinc resource depletion during the advanced stage of the disease.

Serum trace element analysis enables a practical approach for assessing disease progression, and its integration into clinical practice may improve cardiovascular diagnostics.

Future perspectives

Literature lacks reports on the possible role of trace elements in the risk reduction of bioprosthesis degeneration. Based on the current literature and our analysis, we may suspect that the potential influence of zinc, selenium, and chromium deficiency extends not only to native valves and arterial disease. Further studies are necessary in this field.

Based on the literature reporting higher cardiovascular risk in patients with trace element abnormalities, we shall expect a simple strengthened result. Indeed, our analysis showed, e.g., an expected decrease in zinc concentrations. Some results are more puzzling, such as the lack of differences in copper concentration. Notably, our analysis may be influenced by the current modern treatment of hyperlipidemia and heart failure, including statins and flozins, which alter the inflammatory milieu. Moreover, we analyzed patients at the advanced stage of aortic stenosis, with severe calcification, who may present less pronounced inflammation. If these issues may interact with trace element concentration, they need further evaluation. Future research should include long-term and interventional studies to determine if changing trace element levels through diet or medication can slow the progression of aortic stenosis and coronary artery disease. Besides literature reporting an association between trace elements and an increased cardiovascular risk, some analyses highlight the importance of their supplementation in achieving antioxidant effects [60]. In addition, some awareness is raised about the negative effects of overdosing [61]. Thus, our study may serve as a basis for future longitudinal projects concerning the optimal supplementation of essential elements in patients at the early stage of the disease. Studies analyzing tissue samples and advanced molecular markers would be beneficial to clarify how trace metals contribute to calcification in heart valves and blood vessels. This knowledge could lead to new treatments that balance minerals and reduce oxidative stress, potentially slowing disease progression and improving patient outcomes [21].

Limitations

The limitation of our study is the lack of further evaluation of atherosclerosis and/or calcification in other vascular regions, such as carotid arteries or lower limb arteries, which could also be encompassed by the atherosclerotic process. However, the use of blood samples collected proximate to the aortic valve should enhance our analyses.

Moreover, we did not analyze aortic valve or coronary artery disease in terms of trace element content; however, this was related to the transcatheter mode of procedure and the lack of possibility for tissue biopsy. In turn, it enabled the avoidance of changes in serum concentration and inflammatory response related to anesthetic medication and sternotomy.

## 5. Conclusions

Our paper presents data on the blood concentrations of trace and metal elements in patients with isolated aortic stenosis and concomitant coronary artery disease. Blood concentrations of Al, Cr, Mn, Ni, Cu, Se, and Mo are low in patients with aortic stenosis, and this decrease is particularly pronounced in the presence of CAD. These observations suggest that they are transferred to affected tissues and are involved in pathophysiological processes related to disease progression. The results highlight the potential of measuring serum trace elements as biomarkers for disease severity and progression in patients with combined AS and CAD. Future research should focus on longitudinal and intervention studies to understand whether modifying trace element levels could influence disease outcomes. Incorporating molecular and tissue analyses will also help clarify the local roles of these elements in valve and vessel pathology. Such insights could pave the way for new therapeutic approaches aimed at restoring mineral balance and reducing oxidative stress, with the goal of improving patient outcomes.

## Figures and Tables

**Table 1:** Patients’ demographic and clinical data.

	Whole Group (*n* = 53)	CAD Patients (*n* = 26)	Non-CAD Patients (*n* = 27)	*p*	Any Revascularisation (*n* = 15)	Any Revascularisation or Stenosis > 70%/LM > 50% (*n* = 17)
Sex male (*n*,%)	25 (47.2)	13 (50)	12 (44)	0.685	10 (66.7)	10 (58.8)
Age (years, median, Q1–Q3)	78 (75–81)	78 (75.3–80.8)	79 (76–82.5)	0.988	79 (76.5–81.5)	79 (76–81)
EuroScore II (%, median, Q1–Q3)	2.3 (1.7–3.2)	2.5 (1.7–3.5)	2.1 (1.7–2.9)	0.460	2.4 (1.7–3.4)	2.7 (1.7–3.5)
BMI (kg/m^2^, median, Q1–Q3)	28 (24.7–31.2)	27.5 (24.3–33.5)	28 (25.9–30.6)	0.836	28.8 (25.2–34)	28.7 (24.7–33.8)
DM or IGT (*n*,%)	25 (47.2)	13 (50.0)	12 (44.4)	0.685	6 (40.0)	8 (47.1)
HA (*n*,%)	47 (88.7)	23 (88.5)	24 (88.9)	0.961	14 (93.3)	16 (94.1)
COPD (*n*,%)	5 (9.4)	1 (3.9)	4 (14.8)	0.172	1 (6.7)	1 (5.9)
AF (*n*,%)	13 (24.5)	6 (23.1)	7 (25.9)	0.810	3 (20.0)	4 (23.5)
Hyperlipidaemia (*n*,%)	47 (88.7)	24 (92.3)	23 (85.2)	0.413	15 (100)	17 (100)
Previous BAV (*n*,%)	1 (1.9)	0	1 (3.7)	0.322	0	0
PAD (*n*,%)	10 (19.2)	5 (19.2)	5 (19.2)	1.000	2 (13.3)	3 (17.6)
Pacemaker (*n*,%)	4 (7.6)	2 (7.7)	2 (7.4)	0.969	2 (13.3)	2 (11.8)
Kidney disease * (*n*,%)	18 (34.6)	10 (38.5)	8 (30.8)	0.560	5 (33.3)	7 (41.2)
History of smoking (*n*,%)	8 (15.1)	5 (19.2)	3 (11.1)	0.467	1 (6.7)	2 (11.8)
Previous stroke or TIA (*n*,%)	9 (17)	1 (3.9)	8 (29.6)	0.012	1 (6.7)	1 (5.9)
Peak aortic gradient (mmHg, median, Q1–Q3)	83 (72–100)	76 (68.5–93.8)	88 (77–102)	0.096	75 (64–89.5)	75 (60–86)
Mean aortic gradient (mmHg, median, Q1–Q3)	54 (44–63)	50.5 (42.3–60.4)	54 (48.5–64)	0.388	45 (41–59.3)	44 (40–58)
LVEF (%, median, Q1–Q3)	55 (50–60)	57.5 (50–60)	55 (50–60)	0.852	55 (52.5–60)	55 (50–60)
TRPG (mmHg, median, Q1–Q3)	30 (25.5–39.5)	30.3 (25–34.3)	30 (27.5–41)	0.336	26.5 (22.8–33.7)	26.5 (19.8–33.9)

Abbreviations: AF—atrial fibrillation, BAV—balloon aortic valvuloplasty, BMI—body mass index, COPD—chronic obstructive pulmonary disease, DM—diabetes mellitus, GFR—glomerular filtration rate, HA—arterial hypertension, LVEF—left ventricular ejection fraction, LM—left main, PAD—peripheral arterial disease, TIA—transient ischemic attack, TRPG—tricuspid regurgitation peak gradient. * Kidney disease is defined as GFR < 60 mL/min.

**Table 2:** The trace metal concentrations in the blood samples collected from the peripheral artery (P) and in proximity to the aortic valve (A) in patients with isolated aortic stenosis without CAD (Non-CAD group I), patients with aortic stenosis and any degree of CAD (CAD group II), and patients with aortic stenosis and history of coronary revascularization (any revascularization group III), and patients with aortic stenosis and history of coronary revascularization or persisting coronary stenosis > 70% (IV).

	Whole Group (*n* = 53)	Non-CAD (*n* = 27) I	CAD (*n* = 26) II	Any Revascularisation (*n* = 15) III	Any Revascularisation or Stenosis > 70%/LM > 50% (*n* = 17) IV	*p* I vs. II	*p* I vs. III	*p* I vs. IV
P-Li (µg/L, median, Q1–Q3)	1.95 (1.45–2.60)	1.79 (1.40–2.60)	1.98 (1.65–2.38)	1.77 (1.32–2.03)	1.91 (1.45–2.91)	0.90	0.51	0.99
A-Li (µg/L, median, Q1–Q3)	2.06 (1.41–2.60)	2.05 (1.29–2.44)	2.10 (1.69–2.92)	1.75 (1.58–2.15)	1.92 (1.61–2.73)	0.51	0.97	0.71
P-Mg (mg/L, median, Q1–Q3)	33.6 (31.6–37.3	35.6 (31.9–37.2)	33.2 (31.6–37.0)	32.8 (30.1–33.6)	32.8 (28.4–33.6)	0.34	0.09	0.049
A-Mg (mg/L, median, Q1–Q3)	33.1 (30.5–35.9)	32.9 (30.6–35.8)	33.5 (30.3–36.0)	34.2 (30.5–35.8)	33.9 (29.8–35.6)	1.00	0.84	0.84
P-Al (µg/L, median, Q1–Q3)	154.3 (106.2–175.4)	160.9 (153.9–200.5)	113.0 (92.1–163.1)	114.9 (105.4–153.9)	114.9 (107.1–157.6)	0.047	0.05	0.05
A-Al (µg/L, median, Q1–Q3)	153.6 (112.8–171.7)	155.1 (107.5–168.6)	148.1 (113.7–175.6)	137.9 (118.9–169.8)	142.6 (123.7–194.3)	0.83	0.73	1.00
P-Ca (mg/L, median, Q1–Q3)	16.2 (14.5–17.0)	16.6 (16.0–17.3)	15.3 (14.1–16.5)	14.5 (14.1–15.8)	14.5 (14.1–16.3)	0.046	0.03	0.02
A-Ca (mg/L, median, Q1–Q3)	15.4 (13.9–16.6)	15.9 (14.4–16.5)	14.6 (13.5–16.5)	13.6 (12.8–15.2)	13.8 (12.9–16.1)	0.41	0.05	0.16
P-Ti (µg/L, median, Q1–Q3)	206.0 (181.6–225.6)	220.0 (177.5–230.9)	202.7 (187.1–214.9)	194.9 (174.6–210.8)	194.9 (176.7–209.4)	0.25	0.16	0.15
A-Ti (µg/L, median, Q1–Q3)	192.6 (179.2–216.0)	191.9 (177.9–216.3)	198.9 (181.1–209.9)	205.0 (181.3–216.9)	203.8 (178.1–213.8)	0.99	0.79	0.92
P-V (µg/L, median, Q1–Q3)	0.66 (0.56–0.87)	0.69 (0.55–0.85)	0.65 (0.56–0.87)	0.65 (0.55–0.74)	0.65 (0.51–0.81)	0.90	0.68	0.63
A-V (µg/L, median, Q1–Q3)	0.65 (0.52–0.86)	0.65 (0.55–0.87)	0.69 (0.52–0.84)	0.52 (0.50–0.69)	0.53 (0.51–0.76)	0.76	0.16	0.32
P-Cr (µg/L, median, Q1–Q3)	13.8 (7.4–22.1)	13.8 (8.7–21.3)	12.2 (5.6–25.1)	9.7 (4.5–15.7)	8.5 (4.4–15.7)	0.73	0.25	0.12
A-Cr (µg/L, median, Q1–Q3)	14.8 (9.1–27.5)	16.8 (12.3–31.6)	10.3 (5.5–17.4)	5.8 (5.4–13.2)	6.2 (5.4–14.4)	0.07	0.002	0.004
P-Mn (µg/L, median, Q1–Q3)	5.70 (4.02–7.84)	7.01 (4.20–10.35)	5.57 (3.95–6.70)	6.06 (4.66–6.85)	6.1 (4.25–7.24)	0.39	0.65	0.76
A-Mn (µg/L, median, Q1–Q3)	6.05 (3.91–7.35)	7.01 (4.49–8.13)	4.94 (3.37–6.19)	5.00 (3.42–6.05)	5.40 (3.80–6.47)	0.03	0.13	0.13
P-Fe (mg/L, median, Q1–Q3)	423.7 (378.8–452.6)	405.5 (382.3–446.8)	425.8 (379.4–492.9)	425.4 (385.5–460.3)	419.7 (363.8–444.7)	0.40	0.59	0.96
A-Fe (mg/L, median, Q1–Q3)	403.6 (363.0–471.7)	399.8 (389.7–442.4)	417.6 (349.7–474.2)	433.9 (353.9–481.8)	405.7 (351.3–475.1)	0.90	0.67	0.99
P-Co (µg/L, median, Q1–Q3)	0.89 (0.69–0.94)	0.90 (0.70–1.04)	0.85 (0.71–1.03)	0.93 (0.77–1.04)	0.93 (0.75–1.08)	0.88	0.73	0.71
A-Co (µg/L, median, Q1–Q3)	0.84 (0.70–0.94)	0.84 (0.73–0.94)	0.78 (0.59–0.90)	0.75 (0.59–0.90)	0.76 (0.60–0.92)	0.20	0.15	0.30
P-Ni (µg/L, median, Q1–Q3)	20.59 (16.1–28.6)	24.2 (16.8–29.5)	17.6 (14.5–25.8)	16.2 (12.5–21.5)	16.8 (14.1–21.7)	0.20	0.035	0.029
A-Ni (µg/L, median, Q1–Q3)	23.85 (16.2–37.4)	31.5 (16.8–38.8)	21.9 (14.5–27.5)	21.9 (20.8–32.7)	22.0 (21.0–30.3)	0.17	0.49	0.46
P-Cu (µg/L, median, Q1–Q3)	963.3 (870.0–1044.5)	991.4 (915.5–1044.5)	914.2 (844.8–1009.2)	870.0 (777.0–906.9)	876.5 (780.4–921.5)	0.34	0.01	0.03
A-Cu (µg/L, median, Q1–Q3)	917.0 (828.1–1006.8	914.9 (867.9–959.9)	919.6 (751.1–1024.3)	751.1 (706.1–778.3)	752.0 (711.6–900)	0.40	0.009	0.025
P-Zn (µg/L, median, Q1–Q3)	5428.8 (5136.8–6119.0)	5382.3 (4844.9–6040.8)	5466.6 (5202.7–6204.6)	5466.6 (5124.2–5866.7)	5410.6 (5217.5–5717.1)	0.41	0.70	0.76
A-Zn (µg/L, median, Q1–Q3)	5569.2 (4985.5–6271.1)	5554.0 (4969.8–6153.6)	5622.5 (5399.2–6447.7)	5622.5 (5467.9–6447.7)	5545.5 (4979.8–6286.9)	0.49	0.49	0.84
P-As (µg/L, median, Q1–Q3)	0.88 (0.50–1.87)	1.06 (0.54–1.94)	0.85 (0.45–1.62)	0.79 (0.33–1.44)	0.85 (0.34–1.51)	0.50	0.22	0.40
A-As (µg/L, median, Q1–Q3)	0.97 (0.46–1.88)	1.21 (0.47–2.00)	0.73 (0.44–1.78)	0.88 (0.47–1.91)	0.97 (0.54–1.88)	0.43	0.88	0.84
P-Se (µg/L, median, Q1–Q3)	89.7 (76.9–99.7)	94.7 (82.3–101.9)	86.5 (70.9–95.2)	88.0 (68.7–95.2)	82.5 (67.7–93.6)	0.21	0.19	0.008
A-Se (µg/L, median, Q1–Q3)	84.4 (72.6–95.4)	91.0 (74.8–97.9)	79.9 (66.6–86.1)	72.9 (66.6–81.6)	71 (63.6–81.2)	0.007	0.02	0.01
P-Rb (µg/L, median, Q1–Q3)	1103.9 (931.7–1279.5)	1016.9 (894.8–1287.9)	1145.7 (988.6–1232.2)	1106.3 (909.2–1176.6)	1106.3 (882.9–1180.5)	0.44	0.83	0.96
A-Rb (µg/L, median, Q1–Q3)	1105.0 (911.7–1273.9)	1109.2 (902.6–1247.4)	1100.8 (953.1–1315.7)	1062.7 (837.0–1396.8)	1007.9 (808.9–1332.8)	0.82	1.00	0.65
P-Sr (µg/L, median, Q1–Q3)	25.8 (19.1–34.6)	25.8 (19.5–35.8)	24.9 (18.6–30.1)	24.9 (18.6–28.3)	27.3 (19.5–32.5)	0.43	0.35	0.63
A-Sr (µg/L, median, Q1–Q3)	24.8 (19.2–30.7)	26.2 (20.6–30.7)	21.8 (18.9–29.1)	19.6 (19.1–27.7)	22.5 (19.2–28.8)	0.39	0.20	0.36
P-Mo (µg/L, median, Q1–Q3)	4.09 (3.52–4.81)	4.43 (3.75–4.91)	3.83 (3.34–4.80)	3.58 (3.24–3.95)	3.58 (3.19–4.09)	0.25	0.08	0.08
A-Mo (µg/L, median, Q1–Q3)	3.86 (3.55–4.23)	3.88 (3.76–4.28)	3.62 (3.37–4.10)	3.37 (2.69–3.62)	3.45 (2.82–3.85)	0.15	0.008	0.04
P-Cd (µg/L, median, Q1–Q3)	0.33 (0.04–0.48)	0.33 (0.04–0.46)	0.17 (0.07–0.51)	0.10 (0.05–0.35)	0.11 (0.06–0.34)	0.98	0.59	0.56
A-Cd (µg/L, median, Q1–Q3)	0.16 (0.05–0.52)	0.30 (0.04–0.52)	0.10 (0.05–0.47)	0.09 (0.05–0.40)	0.10 (0.05–0.30)	0.85	0.75	0.71
P-Sn (µg/L, median, Q1–Q3)	0.31 (0.15–0.64)	0.18 (0.09–0.88)	0.35 (0.26–0.51)	0.36 (0.28–0.45)	0.36 (0.24–0.47)	0.34	0.52	0.52
A-Sn (µg/L, median, Q1–Q3)	0.55 (0.17–0.98)	0.56 (0.32–0.96)	0.44 (0.15–0.89)	0.50 (0.13–1.11)	0.59 (0.15–1.03)	0.42	0.64	0.70
P-Sb (µg/L, median, Q1–Q3)	26.21 (0.39–32.43)	30.76 (0.40–32.82)	3.04 (0.41–30.28)	2.66 (1.69–27.82)	14.45 (2.28–29.10)	0.32	0.24	0.36
A-Sb (µg/L, median, Q1–Q3)	16.0 (0.5–32.3)	3.2 (0.6–32.0)	29.4 (0.5–32.3)	28.5 (0.4–32.1)	28.9 (0.82–32.2)	0.82	0.88	0.96
P-Cs (µg/L, median, Q1–Q3)	3.3 (2.1–10.4)	3.1 (1.9–5.2)	3.4 (2.4–15.4)	20.3 (5.2–30.5)	8.6 (2.9–30.3)	0.59	0.12	0.29
A-Cs (µg/L, median, Q1–Q3)	4.20 (2.52–12.0)	4.20 (2.74–12.95)	3.81 (2.22–7.09)	4.44 (2.38–28.41)	5.09 (2.56–23.78)	0.61	0.88	0.80
P-Ba (µg/L, median, Q1–Q3)	0.21 (0.04–0.97)	0.41 (0.02–1.48)	0.11 (0.07–0.68)	0.07 (0.03–0.10)	0.08 (0.04–0.15)	1.00	0.62	0.59
A-Ba (µg/L, median, Q1–Q3)	0.18 (0.04–10.14)	0.43 (0.04–19.11)	0.08 (0.04–6.45)	0.05 (0.04–11.25)	0.047 (0.04–10.7)	0.31	0.45	0.42
P-Pb (µg/L, median, Q1–Q3)	0.36 (0.06–15.78)	0.42 (0.05–16.77)	0.16 (0.07–10.85)	0.10 (0.06–9.13)	0.12 (0.07–8.92)	0.64	0.53	0.48
A-Pb (µg/L, median, Q1–Q3)	0.12 (0.03–18.17)	0.38 (0.04–20.88)	0.06 (0.03–13.15)	0.048 (0.03–14.43)	0.055 (0.03–12.05)	0.50	0.71	0.67

Abbreviations: aluminum (Al), arsenic (As), barium (Ba), cadmium (Cd), calcium (Ca), cesium (Cs), cobalt (Co), copper (Cu), coronary artery disease (CAD), chromium (Cr), iron (Fe), lead (Pb), lithium (Li), left main (LM), magnesium (Mg), manganese (Mn), molybdenum (Mo), nickel (Ni), rubidium (Rb), selenium (Se), strontium (Sr), titanium (Ti), vanadium (V), zinc (Zn). Samples designation: Aortic sample (A), Peripheral sample (P).

## Data Availability

All data will be available for three years following the publication, after a reasonable request is presented by e-mail correspondence to the corresponding author.

## Abbreviations

Abbreviations: aortic sample (A), atrial fibrillation (AF), aluminum (Al), arsenic (As), balloon aortic valvuloplasty (BAV), barium (Ba), body mass index (BMI), cadmium (Cd), coronary artery disease (CAD), calcium (Ca), coronary artery bypass grafting (CABG), cesium (Cs), cobalt (Co), chronic obstructive pulmonary disease (COPD), copper (Cu), coronary artery disease (CAD), chromium (Cr), diabetes mellitus (DM), glomerular filtration rate (GFR), impaired glucose tolerance (IGT), iron (Fe), arterial hypertension (HA), left ventricular ejection fraction (LVEF), lithium (Li), magnesium (Mg), manganese (Mn), molybdenum (Mo), nickel (Ni), peripheral sample (P), peripheral arterial disease (PAD), percutaneous coronary intervention (PCI), lead (Pb), rubidium (Rb), selenium (Se), strontium (Sr), titanium (Ti), transient ischemic attack (TIA), tricuspid regurgitation peak gradient (TRPG), vanadium (V), zinc (Zn).

## References

[1] Tomii D., Pilgrim T., Borger M.A., De Backer O., Lanz J., Reineke D., Siepe M., Windecker S. (2024). Aortic Stenosis and Coronary Artery Disease: Decision-Making Between Surgical and Transcatheter Management. Circulation.

[2] Goody P.R., Hosen M.R., Christmann D., Niepmann S.T., Zietzer A., Adam M., Bönner F., Zimmer S., Nickenig G., Jansen F. (2020). Aortic Valve Stenosis: From Basic Mechanisms to Novel Therapeutic Targets. Arter. Thromb. Vasc. Biol..

[3] Wu B., Pei X., Li Z.Y. (2014). How does calcification influence plaque vulnerability? Insights from fatigue analysis. Sci. World J..

[4] Shi X., Gao J., Lv Q., Cai H., Wang F., Ye R., Liu X. (2020). Calcification in Atherosclerotic Plaque Vulnerability: Friend or Foe?. Front. Physiol..

[5] Celeski M., Nusca A., Ciavaroli N.G., Martucciello A., Crisci F., Polito D., Mangiacapra F., Cammalleri V., Melfi R., Gallo P. (2025). Co-Occurrence of Aortic Stenosis and Coronary Artery Disease: Facing Challenges Before, During, and After Transcatheter Aortic Valve Replacement. J. Clin. Med..

[6] Perpétuo L., Barros A.S., Dalsuco J., Nogueira-Ferreira R., Resende-Gonçalves P., Falcão-Pires I., Ferreira R., Leite-Moreira A., Trindade F., Vitorino R. (2022). Coronary Artery Disease and Aortic Valve Stenosis: A Urine Proteomics Study. Int. J. Mol. Sci..

[7] Bartnicka J.J., Blower P.J. (2018). Insights into Trace Metal Metabolism in Health and Disease from PET: “PET Metallomics”. J. Nucl. Med..

[8] Gryszczyńska B., Grupińska J., Kasprzak M.P., Krasiński Z., Iskra M. (2025). Calcium, magnesium and iron blood plasma levels in abdominal aortic aneurysm: The effects of pre-and postoperative treatment. J. Elem..

[9] Urbanowicz T., Hanć A., Olasińska-Wiśniewska A., Rodzki M., Witkowska A., Michalak M., Perek B., Haneya A., Jemielity M. (2022). Serum copper concentration reflect inflammatory activation in the complex coronary artery disease—A pilot study. J. Trace Elem. Med. Biol..

[10] Zeng D., Chen B., Wang H., Xu S., Liu S., Yu Z., Pan X., Tang X., Qin Y. (2025). The mediating role of inflammatory biomarkers in the association between serum copper and sarcopenia. Sci. Rep..

[11] Sun Z., Xu Y., Liu Y., Tao X., Zhou P., Feng H., Weng Y., Lu X., Wu J., Wei Y. (2025). Associations of Exposure to 56 Serum Trace Elements with the Prevalence and Severity of Acute Myocardial Infarction: Omics, Mixture, and Mediation Analysis. Biol. Trace Elem. Res..

[12] Urbanowicz T., Hanć A., Frąckowiak J., Białasik-Misiorny M., Olasińska-Wiśniewska A., Krasińska B., Krasińska-Płachta A., Tomczak J., Kowalewski M., Krasiński Z. (2025). Are Hair Scalp Trace Elements Correlated with Atherosclerosis Location in Coronary Artery Disease?. Biol. Trace Elem. Res..

[13] Cimen Y.A., Taslidere B., Sarikaya U., Demirel M., Acikgoz N., Selek S. (2025). Assessment of oxidative stress and trace element dynamics in acute myocardial infarction and heart failure: A focus on zinc, copper, and thiol dynamics. Clinics.

[14] Tomášek A., Maňoušek J., Kuta J., Hlásenský J., Křen L., Šindler M., Zelený M., Kala P., Němec P. (2023). Metals and Trace Elements in Calcified Valves in Patients with Acquired Severe Aortic Valve Stenosis: Is There a Connection with the Degeneration Process?. J. Pers. Med..

[15] Abdul-Rahman T., Lizano-Jubert I., Garg N., Talukder S., Lopez P.P., Awuah W.A., Shah R., Chambergo D., Cantu-Herrera E., Farooqi M. (2023). The common pathobiology between coronary artery disease and calcific aortic stenosis: Evidence and clinical implications. Prog. Cardiovasc. Dis..

[16] Patel K.P., Michail M., Treibel T.A., Rathod K., Jones D.A., Ozkor M., Kennon S., Forrest J.K., Mathur A., Mullen M.J. (2021). Coronary Revascularization in Patients Undergoing Aortic Valve Replacement for Severe Aortic Stenosis. JACC Cardiovasc. Interv..

[17] Simić A., Hansen A.F., Syversen T., Lierhagen S., Ciesielski T.M., Romundstad P.R., Midthjell K., Åsvold B.O., Flaten T.P. (2022). Trace elements in whole blood in the general population in Trøndelag County, Norway: The HUNT3 Survey. Sci. Total Environ..

[18] Syversen T., Evje L., Wolf S., Flaten T.P., Lierhagen S., Simic A. (2021). Trace Elements in the Large Population-Based HUNT3 Survey. Biol. Trace Elem. Res..

[19] Aggett P.J. (1985). Physiology and metabolism of essential trace elements: An outline. Clin. Endocrinol. Metab..

[20] López-Alonso M., Rivas I., Miranda M. (2025). Trace Mineral Imbalances in Global Health: Challenges, Biomarkers, and the Role of Serum Analysis. Nutrients.

[21] Eroglu B., Eichelmann F., Kuxhausf O., Kipp A.P., Schwerdtle T., Haase H., Schomburg L., Schulze M.B. (2025). Trace elements and risk of diabetes-related vascular complications: Results from the EPIC-Potsdam cohort study. Cardiovasc Diabetol..

[22] Nuermaimaiti K., Li T., Li N., Shi T., Liu W., Abulaiti P., Abulaihaiti K., Gao F. (2025). Vitamin and trace elements imbalance are very common in adult patients with newly diagnosed Celiac disease. Sci. Rep..

[23] Wechselberger C., Messner B., Bernhard D. (2023). The Role of Trace Elements in Cardiovascular Diseases. Toxics.

[24] Fanni D., Gerosa C., Nurchi V.M., Suri J.S., Nardi V., Congiu T., Coni P., Ravarino A., Cerrone G., Piras M. (2021). Trace elements and the carotid plaque: The GOOD (Mg, Zn, Se), the UGLY (Fe, Cu), and the BAD (P, Ca)?. Eur. Rev. Med. Pharmacol. Sci..

[25] Myasoedova V.A., Valerio V., Rusconi V., Bertolini F., Massaiu I., Pirola S., Gripari P., Mantegazza V., Cannata F., Stankowski K. (2025). Systemic inflammation and fibrocalcific remodeling in aortic stenosis: The interplay of Lipoprotein(a), sex, and valve morphology. J. Transl. Med..

[26] Nosrati R., Kheirouri S., Ghodsi R., Ojaghi H. (2019). The effects of zinc treatment on matrix metalloproteinases: A systematic review. J. Trace Elem. Med. Biol..

[27] Chen B., Yu P., Chan W.N., Xie F., Zhang Y., Liang L., Leung K.T., Lo K.W., Yu J., Tse G.M.K. (2024). Cellular zinc metabolism and zinc signaling: From biological functions to diseases and therapeutic targets. Signal Transduct. Target. Ther..

[28] Starcher B.C., Hill C.H., Madaras J.G. (1980). Effect of zinc deficiency on bone collagenase and collagen turnover. J. Nutr..

[29] Meng H., Ruan J., Chen Y., Yan Z., Meng X., Li X., Liu J., Mao C., Yang P. (2022). Serum Zinc Ion Concentration Associated with Coronary Heart Disease: A Systematic Review and Meta-Analysis. Cardiol. Res. Pract..

[30] Banik S., Ghosh A. (2022). Zinc status and coronary artery disease: A systematic review and meta-analysis. J. Trace Elem. Med. Biol..

[31] Huang L., Liu Y., Yu L., Cheng A., Cao J., Wang R., Liu Y., Song S., Zhao W., Liu Q. (2025). Association of metal elements deposition with symptomatic carotid artery stenosis and their spatial distribution in atherosclerosis plaques. Metallomics.

[32] Mantsou A., Pitou M., Papachristou E., Papi R.M., Lamprou P., Choli-Papadopoulou T. (2021). Effect of a Bone Morphogenetic Protein-2-derived peptide on the expression of tumor marker ZNF217 in osteoblasts and MCF-7 cells. Bone Rep..

[33] Wang Y.M., Feng L.S., Xu A., Ma X.H., Zhang M.T., Zhang J. (2024). Copper ions: The invisible killer of cardiovascular disease (Review). Mol. Med. Rep..

[34] Fang X., Weintraub N.L., Rios C.D., Chappell D.A., Zwacka R.M., Engelhardt J.F., Oberley L.W., Yan T., Heistad D.D., Spector A.A. (1998). Overexpression of human superoxide dismutase inhibits oxidation of low-density lipoprotein by endothelial cells. Circ Res..

[35] Tribble D.L., Gong E.L., Leeuwenburgh C., Heinecke J.W., Carlson E.L., Verstuyft J.G., Epstein C.J. (1997). Fatty streak formation in fat-fed mice expressing human copper-zinc superoxide dismutase. Arter. Thromb. Vasc. Biol..

[36] Li J., Yi L., Zhang L., Shen L., Lu Y., Wang H., Chen X., Kou Y., Wang Y., Ma R. (2025). Association of Copper with Atherosclerosis and Treatment Strategies. J. Cardiovasc. Transl. Res..

[37] Chen Z., Li Y.Y., Liu X. (2023). Copper homeostasis and copper-induced cell death: Novel targeting for intervention in the pathogenesis of vascular aging. Biomed. Pharmacother..

[38] Zhao H., Mei K., Hu Q., Wu Y., Xu Y., Qinling Yu P., Deng Y., Zhu W., Yan Z., Liu X. (2024). Circulating copper levels and the risk of cardio-cerebrovascular diseases and cardiovascular and all-cause mortality: A systematic review and meta-analysis of longitudinal studies. Environ. Pollut..

[39] Muñoz-Bravo C., Soler-Iborte E., Lozano-Lorca M., Kouiti M., González-Palacios Torres C., Barrios-Rodríguez R., Jiménez-Moleón J.J. (2023). Serum copper levels and risk of major adverse cardiovascular events: A systematic review and meta-analysis. Front. Cardiovasc. Med..

[40] Dziedzic E.A., Tuzimek A., Gąsior J.S., Paleczny J., Junka A., Kwaśny M., Dąbrowski M., Jankowski P. (2022). Investigation on the Association of Copper and Copper-to-Zinc-Ratio in Hair with Acute Coronary Syndrome Occurrence and Its Risk Factors. Nutrients.

[41] Chen A., Li G., Liu Y. (2015). Association between copper levels and myocardial infarction: A meta-analysis. Inhal. Toxicol..

[42] Morvaridzadeh M., Estêvão M.D., Qorbani M., Heydari H., Hosseini A.S., Fazelian S., Belančić A., Persad E., Rezamand G., Heshmati J. (2022). The effect of chromium intake on oxidative stress parameters: A systematic review and meta-analysis. J. Trace Elem. Med. Biol..

[43] Guallar E., Jiménez F.J., van ‘t Veer P., Bode P., Riemersma R.A., Gómez-Aracena J., Kark J.D., Arab L., Kok F.J., Martín-Moreno J.M. (2005). Low toenail chromium concentration and increased risk of nonfatal myocardial infarction. Am. J. Epidemiol..

[44] Kosmopoulos M., Drekolias D., Zavras P.D., Piperi C., Papavassiliou A.G. (2019). Impact of advanced glycation end products (AGEs) signaling in coronary artery disease. Biochim. Biophys. Acta Mol. Basis Dis..

[45] Kopytek M., Ząbczyk M., Mazur P., Undas A., Natorska J. (2020). Accumulation of advanced glycation end products (AGEs) is associated with the severity of aortic stenosis in patients with concomitant type 2 diabetes. Cardiovasc. Diabetol..

[46] Guadalupe Hernández J., Thangarasu P. (2024). Chromium Complex of Macrocyclic Ligands as Precursor for Nitric Oxide Release: A Theoretical Study. Chemphyschem.

[47] Erdely A., Antonini J.M., Young S.H., Kashon M.L., Gu J.K., Hulderman T., Salmen R., Meighan T., Roberts J.R., Zeidler-Erdely P.C. (2014). Oxidative stress and reduced responsiveness of challenged circulating leukocytes following pulmonary instillation of metal-rich particulate matter in rats. Part. Fibre Toxicol..

[48] Zhang Y., Roh Y.J., Han S.J., Park I., Lee H.M., Ok Y.S., Lee B.C., Lee S.R. (2020). Role of Selenoproteins in Redox Regulation of Signaling and the Antioxidant System: A Review. Antioxidants.

[49] Brigelius-Flohé R., Banning A., Schnurr K. (2003). Selenium-dependent enzymes in endothelial cell function. Antioxidants Redox Signal..

[50] Rashtchizadeh N., Karimi P., Dehgan P., Salimi Movahed M. (2015). Effects of Selenium in the MAPK Signaling Cascade. J. Cardiovasc. Thorac. Res..

[51] Orian L., Flohé L. (2021). Selenium-Catalyzed Reduction of Hydroperoxides in Chemistry and Biology. Antioxidants.

[52] Lawrence T. (2009). The nuclear factor NF-kappaB pathway in inflammation. Cold Spring Harb. Perspect. Biol..

[53] Tak P.P., Firestein G.S. (2001). NF-kappaB: A key role in inflammatory diseases. J. Clin. Investig..

[54] Klauzen P., Basovich L., Shishkova D., Markova V., Malashicheva A. (2024). Macrophages in Calcific Aortic Valve Disease: Paracrine and Juxtacrine Disease Drivers. Biomolecules.

[55] Scioli M.G., Storti G., D’Amico F., Rodríguez Guzmán R., Centofanti F., Doldo E., Céspedes Miranda E.M., Orlandi A. (2020). Oxidative Stress and New Pathogenetic Mechanisms in Endothelial Dysfunction: Potential Diagnostic Biomarkers and Therapeutic Targets. J. Clin. Med..

[56] Detopoulou P., Letsiou S., Nomikos T., Karagiannis A., Pergantis S.A., Pitsavos C., Panagiotakos D.B., Antonopoulou S. (2023). Selenium, Selenoproteins and 10-year Cardiovascular Risk: Results from the ATTICA Study. Curr. Vasc. Pharmacol..

[57] Nyström-Rosander C., Lindh U., Thelin S., Lindquist O., Friman G., Ilbäck N.G. (2002). Trace element changes in sclerotic heart valves from patients undergoing aortic valve surgery. Biol. Trace Element Res..

[58] Lis G.J., Czapla-Masztafiak J., Kwiatek W.M., Gajda M., Jasek E., Jasinska M., Czubek U., Borchert M., Appel K., Nessler J. (2014). Distribution of selected elements in calcific human aortic valves studied by microscopy combined with SR-μXRF: Influence of lipids on progression of calcification. Micron.

[59] Al-Taesh H., Çelekli A., Sucu M., Taysi S. (2021). Trace elements in patients with aortic valve sclerosis. Ther. Adv. Cardiovasc. Dis..

[60] Jenkins D.J.A., Kitts D., Giovannucci E.L., Sahye-Pudaruth S., Paquette M., Blanco Mejia S., Patel D., Kavanagh M., Tsirakis T., Kendall C.W.C. (2020). Selenium, antioxidants, cardiovascular disease, and all-cause mortality: A systematic review and meta-analysis of randomized controlled trials. Am. J. Clin. Nutr..

[61] Kuria A., Tian H., Li M., Wang Y., Aaseth J.O., Zang J., Cao Y. (2021). Selenium status in the body and cardiovascular disease: A systematic review and meta-analysis. Crit. Rev. Food Sci. Nutr..

